# Association of Agenesis of the Dorsal Pancreas With *HNF1B* Heterozygote Mutation: A Case Report

**DOI:** 10.3389/fendo.2021.640006

**Published:** 2021-10-15

**Authors:** Mei Guo, Qinqin Xu, Xuefeng Yu, Qin Yang, Shiying Shao

**Affiliations:** ^1^ Division of Endocrinology, Tongji Hospital, Huazhong University of Science & Technology, Wuhan, China; ^2^ Division of Endocrinology, Qianjiang Central Hospital of Hubei Province, Qianjiang Hospital Affiliated to Renmin Hospital of Wuhan University, Qianjiang Clinical Medical College, Health Science Center, Yangtze University, Qianjiang, China; ^3^ Branch of National Clinical Research Center for Metabolic Diseases, Wuhan, China; ^4^ Division of Pathology, Tongji Hospital, Huazhong University of Science & Technology, Wuhan, China

**Keywords:** agenesis of the dorsal pancreas, diabetes, hepatocyte nuclear factor 1B, diabetes ketoacidosis, case report

## Abstract

**Background:**

Agenesis of the dorsal pancreas (ADP) is a rare disease, the pathogenic mechanism of which is partially related to variants of hepatocyte nuclear factor 1B (*HNF1B*) gene.

**Case Presentation:**

We report a case of ADP, which presented with acute ketoacidosis, hyperuricemia, and liver dysfunction. In this case, the *HNF1B* score was estimated as 16 and a heterozygous variant of *HNF1B* in exon 2 (c.513G>A-p.W171X) was identified through gene sequencing.

**Conclusions:**

A good understanding of the clinical comorbidities of ADP is essential for avoiding missed diagnosis to a great extent. Moreover, estimation of *HNF1B* score is recommended before genetic testing.

## Background

Agenesis of the dorsal pancreas (ADP) is an extremely rare congenital malformation, which is featured by the absence of corpus and cauda of the pancreas. The first case of ADP was described in 1911 and 134 cases have been reported up till 2021 (1–8). Genes, whose mutation may cause pancreatic agenesis, include GATA binding protein 6 (*GATA6*), insulin promoter factor-1 (*IPF1*), pancreas transcription factor 1alpha (*PTF1A*), hepatocyte nuclear factor 1A (*HNF1A*), and hepatocyte nuclear factor 1B (*HNF1B*). Howbeit, detailed pathogenesis and molecular mechanisms of this disease have not been thoroughly understood.

Although patients with ADP could present a wide variety of clinical comorbidities (9), only three cases were reported to be accompanied with diabetes ketoacidosis (DKA) (3, 10, 11). Here we report one case of a 21-year-old man with multiple clinical manifestations of ADP including DKA, hypomagnesemia, hyperuricemia, and asymptomatic liver dysfunction; other abnormalities such as renal cyst were present. It is assumed that the above manifestations are associated with the heterozygote variant in the *HNF1B* gene.

## Case Presentation

A 21-year-old Chinese man was hospitalized in August 2019 due to the symptoms of thirst, polydipsia and fatigue (**Figure S1**). The laboratory findings showed a random blood glucose level of 65.14mmol/L along with positive urine ketone and acidemia. This patient was diagnosed as DKA and received insulin infusion for glucose control and fluid replacement to correct dehydration. After 8 days of treatment, he was transferred to our hospital for further clinical assessment. The patient did not present personal or family history of diabetes, pancreatitis, abdominal pain, or other diseases.

**Figure 1:**
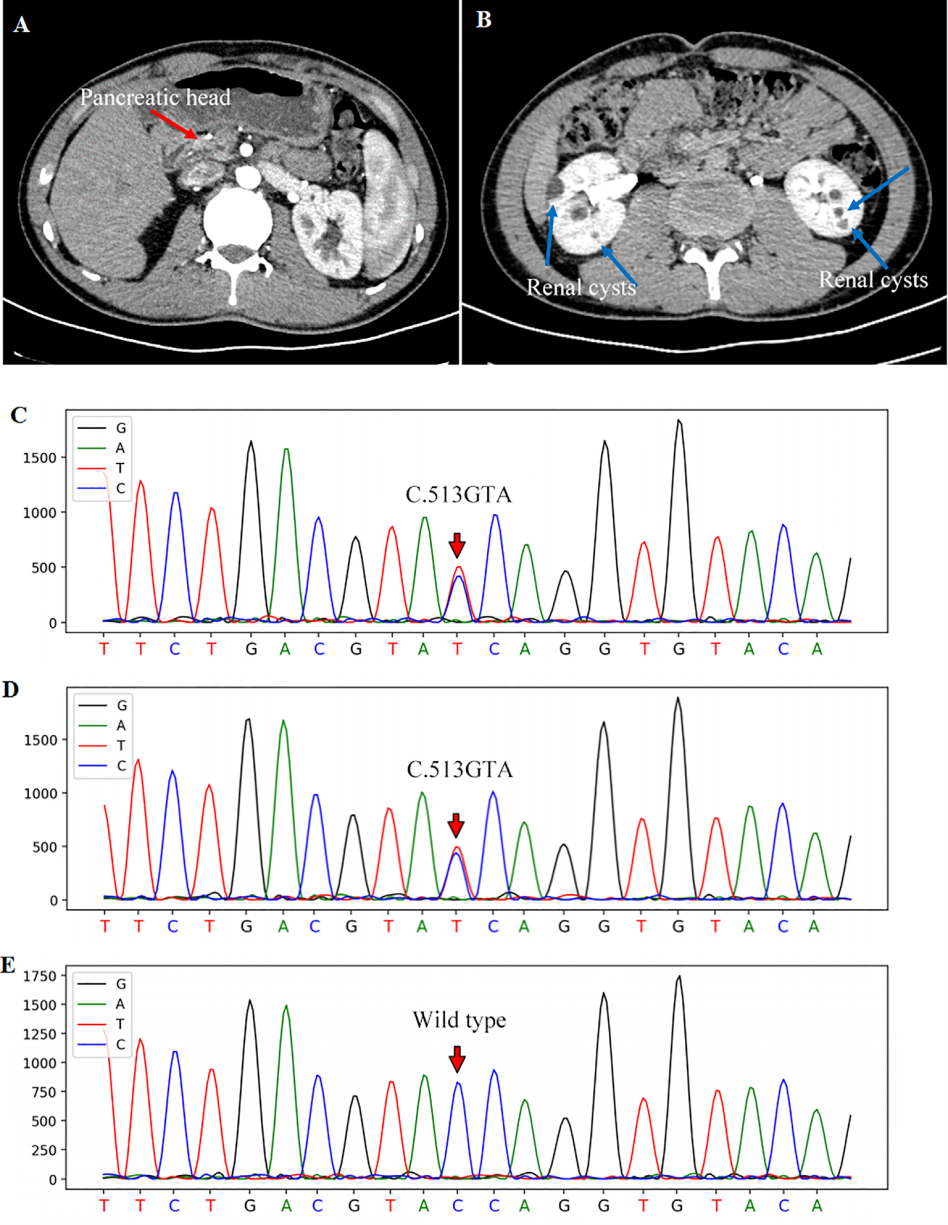
Contrast abdominal CT showed the pancreatic head [**(A)**, red arrow] with the absence of the pancreatic body and tail. Renal cortical cysts on bilateral kidneys were pointed out with blue arrows **(B)**. The gene sequencing showed a heterozygous mutation of *HNF1B* gene in exon 2 (c.513G>A) of the patient **(C)** and his father **(D)**. The sequencing of his mother was wild-type **(E)**.

On physical examination, the patient was lean in shape with body mass index (BMI) 19.0 kg/m^2^. His vital signs were normal with blood pressure 121/74mmHg, body temperature 36.8°C and heart rate 78 beats/minute. Physical examination disclosed no obvious abnormality with negative abdominal tenderness and rebound pain. The patient had no clinical manifestation of dehydration and 24-hour urine volume was around 1500-2000mL. Fasting blood glucose (FBG) on admission was 13.26 mmol/L with glycosylated hemoglobin (HbA1c) 11.9% and C peptide 0.17ng/mL, indicating a poor β-cell function. Howbeit, tests for diabetes-related autoantibodies were all negative, which do not support the diagnosis of autoimmune diabetes. Moreover, the diabetic complications were screened and no signs of diabetic retinopathy, neuropathy or nephropathy was identified. Liver transaminases significantly increased in this case while blood tests for viral hepatitis and autoimmune liver diseases were negative. To identify the cause of abnormal liver function, the abdominal contrast computer tomography (CT) was performed and the result revealed a regular biliary tree, agenesis of the pancreatic body and tail with a normal pancreatic head (**Figure 1A**), and multiple renal cortical cysts in bilateral kidneys (**Figure 1B**). The patient refused further examinations of endoscopic retrograde cholangiopancreatography (ERCP) and magnetic resonance cholangiopancreatography (MRCP) due to a high medical expense. In addition, hypomagnesia (magnesium 0.41mmol/L) and hyperuricemia (uric acid 445μmol/L) were identified in this case (**Table 1**). Based on the above findings, the patient was diagnosed as ADP with diabetes, renal cortical cysts, increased liver transaminases, hypomagnesia, and hyperuricemia.

**Table 1:** Laboratory examinations at the time of admission and follow-up.

		On admission	On discharge	3-month follow-up	21-month follow-up
**Blood Routine**	WBC (3.50-9.50×10^9^/L)	5.80×10^9^/L	/	/	/
	RBC (4.30-5.80×10^12^/L)	4.65×10^12^/L	/	/	/
	PLT (125.0-350.0×10^9^/L)	186.0×10^9^/L	/	/	/
**Liver function**	ALT (<41U/L)	288U/L	71U/L	35U/L	/
	AST (<41U/L)	150U/L	28U/L	21U/L	/
**Renal function**	BUN (3.1-8.0mmol/L)	3.90mmol/L	/	/	/
	Cr (59-104µmol/L)	78µmol/L	/	/	/
	eGFR (>90 mL/min/1.73m^2^)	122.7mL/min/1.73m^2^	/	/	/
	Uric acid (202.3-416.5μmol/L)	445μmol/L	/	438μmol/L	466μmol/L
	UACR (<30.0ug/mg)	4.7ug/mg	/	/	/
**Urine routine**	Leukocytes	(-)	/	/	/
	Erythrocyte	(-)	/	/	/
	Urine protein	(-)	/	/	/
	Ketone bodies	(-)	/	/	/
**Electrolyte**	Potassium (3.50-5.10mmol/L)	4.14mmol/L	/	/	/
	Sodium (136-145mmol/L)	138.9mmol/L	/	/	/
	Calcium (2.15-2.50mmol/L)	2.35mmol/L	/	/	/
	Magnesium (0.66-1.07mmol/L)	0.41mmol/L	0.71 mmol/L	0.78 mmol/L	/
**Blood lipid**	Triglyceride (<1.70mmol/L)	3.02 mmol/L	/	/	/
	Total cholesterol (<5.18mmol/L)	4.47mmol/L	/	/	/
**Glycemic profile**	Fasting glucose (4.11-6.05mmol/L)	13.26mmol/L	5.4mmol/L	5.9mmol/L	6.3mmol/L
	HbA1c (4.27-6.07%)	11.9%	/	6.8%	5.7%
	C peptide (0.3-3.73ng/mL)	0.17ng/mL	/	0.43ng/mL	0.67
**Autoantibodies in Diabetes**	GAD (0-10IU/mL)	0.83IU/mL	/		/
	IAA (0-1.0COI)	0.14COI	/		/
	ICA (0-1.0COI)	0.08COI	/		/
**AMY and LPS**	AMY (13-53U/L)	8U/L	/		/
	LPS (13.00-60.00IU/L)	10.2IU/L	/		/
**Viral hepatitis**	HBsAg (<0.05IU/ml)	0.00IU/mL	/		/
	HCAg (<1.0S/CO)	0.03S/CO	/		/
**Autoimmune liver disease**	ANA	(-)	/		/
	SMA	(-)	/		/
	LKM1	(-)	/		/
	SLA	(-)	/		/
**Thyroid function**	TSH (0.27-4.2 µIU/mL)	4.07µIU/mL	/		/
	FT4 (9.32-17.09ng/L)	13.41ng/L	/		/
	FT3 (2.0-4.4pg/mL)	2.99pg/mL	/		/

ANA, antinuclear antibodies; AMY, amylase; ALT, Alanine aminotransferase; AST, Aspertate aminotransferase; BUN, blood urea nitrogen; Cr, creatinine; eGFR, estimated glomerular filtration rate; FT4, free thyroxine; FT3,free triiodothyronine; GAD, Glutamate decarboxylase antibody; HbA1c, glycosylated hemoglobin; HBsAg, hepatitis B virus surface antigen; HCAg, hepatitis C antibody; IAA, insulin autoantibody; ICA, insular cellular antibody; LKM1, Anti-liver and kidney microsomes antibody type 1; LPS, lipase; PLT, platelet; Hb, hemoglobin; RBC, red blood cell; SLA, Anti-soluble liver antigen antibody; SMA, smooth muscle antibody; TSH, thyroid-stimulating hormone; UA, Uric acid; UACR, urinary albumin/creatinine ratio; WBC, white blood cell;/, not detect.

To further explore the etiology, genomic DNA was extracted from the peripheral blood leukocytes of this patient and his parents for the all-exon gene sequencing (Second generation, Kindstar global, ABI, 3730xl, USA). A heterozygous variant of c.513G>A-p.W171X in exon 2 of *HNF1B* gene was found in this patient (**Figure 1C**), which was further verified by first generation DNA sequencing. Importantly, his father also showed a heterozygous variant in *HNF1B* gene (**Figure 1D**), while his mother showed a wild-type genotype (**Figure 1E**). Thus, the patient’s father underwent a comprehensive evaluation including blood biochemical tests, glucose and HbA1c levels, ultrasound of abdomen and kidney. All these findings were normal.

Hence, insulin subcutaneous pump was set up due to the glucose excursion in this patient and daily dose was tittered to a total dose of 46IU per day. After 5-day treatment with insulin infusion, the FBG was controlled between 5-6mmol/L and 2-hour postprandial blood glucose (PBG) 5-7mmol/L. Thereafter, the treatment was changed to the combination of glargine and insulin aspartate and the patient agreed to this therapeutic regimen. In addition, the abnormal liver function was considered to be related to *HNF1B* variant as well (3). After the treatment with magnesium isoglycyrrhizinate and polyene phosphatidylcholine, the alanine aminotransferase (ALT) descended from 288U/L to 71U/L and aspertate aminotransferase (AST) from 150U/L to 28U/L. Besides, low-dose magnesium was supplemented orally and the blood level of magnesium reached normal range on discharge (**Table 1**).

The follow-up visits were performed every 3-6 months. The most recent return visit was performed on May 9^th^, 2021 (**Figure S1**). The blood glucose was well controlled with fasting C peptide 0.67 ng/mL and HbA1c 5.7% (**Table 1**).

## Discussion

It is known that pancreas develops from the dorsal and ventral pancreatic buds on opposite sides of the foregut. The former forms the tail and body of pancreas while the latter forms the posterior part of the head (1). ADP occurs during fetal development when the dorsal bud fails to form the corpus and cauda (1).

As shown in **Table 2**, clinical comorbidities of ADP include diabetes, pancreatitis, elevated liver enzymes, gallstones, abdominal pain, and various visceral organ malformations, such as kidney cysts, reproductive tract malformation, and so on (1–6, 8, 12–14). Besides, various tumors can also be identified, such as pancreatic tumor, hepatobiliary tumor, intramesocolic tumor, endometrial stromal sarcoma, and carcinoma of tongue (1–3). These concomitant manifestations of ADP mentioned in **Table 2** are not meant to cover all identified symptoms in all reported cases, but rather to demonstrate that various clinical comorbidities may present in patient with ADP. As reported in **Table 2**, there are 68 reported cases of ADP (approximately 50%) accompanied with hyperglycemia, which partly results from the lack of islets (3, 10, 11). It is known that the majority of islets are located in the dorsal pancreas. The decreased β-cell mass and limited capacity of *in vivo* replication lead to insulin insufficiency and the resultant disorder of glucose metabolism (1). The high insulin dosage, which is required to control glucose level of the patient in this case, is consistent with an insulin shortage caused by the loss of pancreatic tissue. Although β-cell dysfunction is often indicative of hyperglycemia, there are only three studies reporting a correlation between ADP and DKA (3, 10, 11), indicating that degrees of β-cell dysfunction are varied among patients with ADP. In addition, abdominal pain (54 cases, 40%), pancreatitis (22 cases, 17%), and renal cysts (14 cases, 10%) are also common clinical manifestations in patients with ADP.

**Table 2:** Concomitant manifestations and *HNF1B* variants of ADP.

Organ	Manifestations	Features	Reported case number (N = 134)	*HNF1B* variants	*HNF1B* score	Reference
Pancreas	Diabetes/prediabetes	DKA	3	/	/	(3, 10, 11)
		Hyperglycemia	65	/	/	(1–4, 7, 8)*
				p.R137_K161del	10	(12)
				p.R137_K161del	8	(12)
				p.R137_K161del	12	(12)
				p.F148L	26	(12)
	Pancreatic tumor		16	/	/	(1–3)*
	Exocrine dysfunction	Pancreatitis	22	/	/	(1–3)*
		Pancreatic enzyme deficiency	11			(1, 3)*
Hepatobiliary system	Elevated liver enzyme	ALT, AST elevation	9	/	/	(1–3)*
	Cholelithiasis	Gallstones	7	/	/	(1–3)*
		Choledocholitiasis	1	/	/	
	Hepatobiliary tumor	Bile duct schwannoma	1	/	/	(1–3)*
		Ampullary carcinoma	2	/	/	
	Hepatobiliary malformation	Choledochal cyst	2	/	/	(1–3)*
Kidney	Renal malformation	Renal cysts	14	/	/	(1)*
				p.R137_K161del	10	(12)
				p.R137_K161del	12	(12)
				p.F148L	26	(12)
				p.F148L	10	(12)
				Heterozygous whole gene deletion	12	(13)
				p.T170P	22	(14)
				c.544+1G>T	22	(14)
				p.R177X	22	(14)
				p.R276X	28	(14)
				17q12 deletion	19	(14)
				17q12 deletion	18	(14)
				17q12 deletion	13	(14)
				17q12 deletion	8	(5)
		Polycystic kidney	1	/	/	(3)*
		Renal agenesis	1	/	/	(3)*
		Malrotated kidney	1	/	/	(3)*
		Pancake kidney	1	/	/	(3)*
	Tubular dysfunction	Hyperuricemia	2	/	/	(3)*
		Hypomagnesemia	0	/	/	(9)
Spleen	Spleen malformation	Polysplenia	14	/	/	(1–3, 6)*
		Enlarged ectopic spleen	1	/	/	(1)*
Gastrointestinal tract	Intestinal obstruction		2	/	/	(1, 2)*
	Duodenal malrotation		4	/	/	(1, 2)*
	Intramesocolic tumor		1	/	/	(2)*
Cardiovascular system	Cardiovascular malformation	Atrial and ventricular septal defects	2	/	/	(1)*
		Sinus venosus atrial defect	1	/	/	(1)*
		Pulmonary stenosis	1	/	/	(1)*
		Tetralogy of Fallot	1	/	/	(1)*
		Aortic coarctation	1	/	/	(1)*
		Transposition of great vessels	1	/	/	(1)*
Genital tract	Uterus malformation	Bicornuate uterus	2	/	/	(1, 2)*
		Enlarged uterus	1	/	/	
	Vaginal atresia		1	/	/	(2)*
	Ovarian cyst		1	/	/	(2)*
	Uterus tumor	Endometrial stromal sarcoma	1	/	/	(2)*
Lung	Pulmonary infection	Pulmonary tuberculosis	1	/	/	(1)*
		Bronchopneumonia	2	/	/	
Other	Abdominal pain		54	/	/	(1–5, 7)*
	Carcinoma of tongue		1	/	/	(3)*
	Hypothyroidism		1	/	/	(1)*

/, not assessed; *, Case(s) included in the listed review(s); ALT, Alanine aminotransferase; AST, Aspertate aminotransferase; DKA, Diabetic ketoacidosis.

Clissold et al. summarized a *HNF1B* gene-associated renal and extra-renal clinical spectrum (9). The majority of concomitant manifestations listed in **Table 2** among patients with ADP overlapped with *HNF1B* spectrum (9). It is well known that *HNF1B* is a transcription factor that plays an essential role in early development and organogenesis of several organs including pancreas, liver, lung, kidney, urogenital tract, and parathyroid gland. Early reports demonstrated an association between *HNF1B* mutations and maturity-onset diabetes of the young (MODY). These patients often presented with renal cysts and were initially considered as renal cysts and diabetes (RCAD) syndrome (15). It is now evident that mutation of this gene results in a panel of *HNF1B*-related manifestations. There were plenty of patients presenting with gout as initial symptom. In addition, some patients displayed hypomagnesemia, which may mimic Gitelman syndrome (16). Howbeit, there are no published guidelines on the screening of potential associated abnormalities in ADP patients.

A limited number of literature and studies reported the association of ADP with *HNF1B* mutation. This gene is located on chromosome 17q12, several mutation forms of which have been identified including deletion (small insertion-deletion or whole-gene deletion), nonsense, missense, frame-shift and splicing mutations (9). **Table 2** listed the reported *HNF1B* variants in patients with ADP and only one case displayed whole gene deletion. Most of these mutations are familial. Accordingly, *HNF1B*-associated diseases are generally considered to be inherited in an autosomal dominant manner. Nevertheless, spontaneous mutations (either site mutations or whole-gene deletions) occur as well (9). Recently, a Japanese cohort study recruited 33 cases with heterozygous variants in *HNF1B* gene or whole-gene deletions and only 7 cases presented with pancreatic malformations (14), indicating that a possible penetrance of *HNF1B* mutation is around 21%. However, there were regional limitations in this study and the sample size was small. Larger scale investigations with multiple countries and regions are necessary to obtain a more comprehensive understanding on the penetrance. In our case, gene sequencing identified a heterozygous variant in exon 2 (c.513G>A-p.W171X) in *HNF1B* gene. The patient’s father also displayed the heterozygous variant, suggesting that this variant was probably paternally inherited. In addition, this mutation site and nucleotide change have also previously been reported by Heidet et al. (17) and recorded in the Human Gene Mutation Database (HGMD, https://www.hgmd.cf.ac.uk). Interestingly, although carrying the same genotype, the patient in that case displayed only bilateral cortical cysts without ADP or other *HNF1B*-related manifestations (17).

No genotype-phenotype correlation has been identified in previous studies. Yorifuji et al. reported two siblings with S148W missense mutation in *HNF1B* (18). Howbeit, they showed different phenotypes: one displayed neonatal diabetes without renal disease whereas his brother suffered from severe renal disease but without diabetes (18). The patient in our case exhibited a diversity of *HNF1B*-related manifestations, but his father did not present glucose metabolism disorder and other *HNF1B*-related diseases. Due to the lack of genotype-phenotype correlations among the various types of *HNF1B* gene mutations, it is uncertain whether the offspring of this patient will inherit some or all the *HNF1B*-related clinical phenotypes. The reasons for phenotypic variation in *HNF1B*-associated disease remain poorly understood. It is uncertain whether such variation is attributed to the functional effects of different mutation sites and forms of *HNF1B* mutation. In addition, other genetic or environmental factors may also play a considerable role in the pattern and severity of *HNF1B*-related clinical features. The underlying mechanisms of genotype-phenotype correlation need to be further studied.

Recently, a *HNF1B* score system was developed to select patients for gene analysis based on clinical, imaging, and biological variables (19). The abnormalities of kidney, genitalia and pancreas obtain the highest score, followed by other parameters including positive family history, antenatal renal abnormalities, hypomagnesaemia, early-onset gout, and abnormal liver function of unknown origin. Using this *HNF1B* score system, the authors determined an optimal cutoff threshold of 8 to rule out *HNF1B* analysis with a sensitivity of 98.2% and a specificity of 41.1% (19). We further summarized *HNF1B* scores for ADP patients in **Table 2**, all of which exceeded 8 with the average score value of 16.35. In current case, the *HNF1B* score reached 16. Thus, we recommend an evaluation of the *HNF1B* score prior to *HNF1B* sequencing for a higher accuracy and simplicity. Regular monitoring and re-evaluation of the *HNF1B* score in extended family members of each propositus would be necessary.

The limitation of the current case is the failure on performing ERCP or MRCP to further confirm the agenesis of the pancreatic body and tail. Despite a conclusion on the findings of pancreatic agenesis by 3 experienced radiologists, a combination of ERCP or MRCP with CT could improve the accuracy and sensitivity of the diagnosis.

## Conclusion

It has been identified that ADP could be complicated with multiple abnormalities in kidney, liver, and genitalia. The associated phenotypic spectrum is still expanding. A better understanding of the phenotypic spectrum of *HNF1B*-related ADP is of significance for clinicians to make a comprehensive evaluation of this disease and to avoid missed diagnosis of possible malignant tumors. There is no established guideline for the treatment of ADP. We suggest that only symptomatic patients should be treated. Moreover, the estimation of *HNF1B* score is recommended, which helps clinicians to determine the necessity of genetic testing.

## Ethics Statement 

Written informed consent was obtained from the individual(s) for the publication of any potentially identifiable images or data included in this article.

## Author Contributions

Study concept and critical revisions: SS, XY, and QY. Paper preparation and data collection: MG. and QX. All authors contributed to the article and approved the submitted version.

## Funding

This study was supported by the grants from the Bethune·Merck Diabetes Research Fund (grant number 2018 to SS, 2018) and Cardiac rehabilitation and metabolic therapy research fund (grant number 2018 to SS, 2018).

## Conflict of Interest

The authors declare that the research was conducted in the absence of any commercial or financial relationships that could be construed as a potential conflict of interest.

## Publisher’s Note

All claims expressed in this article are solely those of the authors and do not necessarily represent those of their affiliated organizations, or those of the publisher, the editors and the reviewers. Any product that may be evaluated in this article, or claim that may be made by its manufacturer, is not guaranteed or endorsed by the publisher.

## Supplementary Material

Supplementary Figure 1 Timeline of the treatment and follow-up visits.
