# Supplementary figures and images for: Association of Agenesis of the Dorsal Pancreas With HNF1B Heterozygote Mutation: A Case Report
Source: Front Endocrinol (Lausanne). 2021 Oct 15;12:640006. doi: 10.3389/fendo.2021.640006 (PMC8554068; doi:10.3389/fendo.2021.640006)

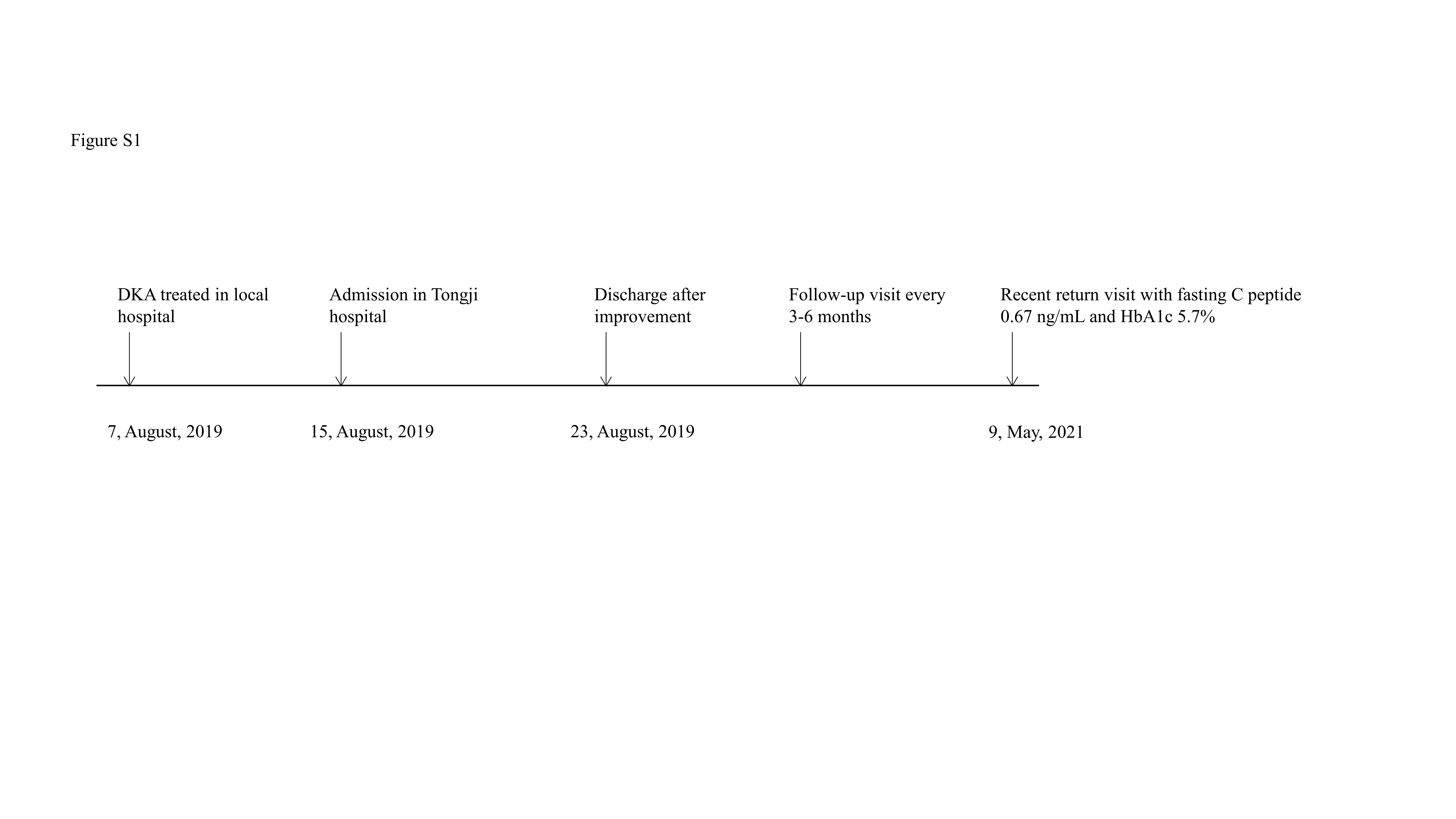

Supplement: Supplementary Figure 1 — Timeline of the treatment and follow-up visits. [file Image_1.tif]
